# High risk of plant invasion in the understory of eucalypt plantations in South China

**DOI:** 10.1038/srep18492

**Published:** 2015-12-21

**Authors:** Dongmei Jin, Yong Huang, Xi-Le Zhou, Bin Chen, Jinshuang Ma, Yue-Hong Yan

**Affiliations:** 1 Shanghai Chenshan Plant Science Research Center, Chinese Academy of Sciences/Shanghai Chenshan Botanical Garden, Shanghai 201602, China; 2 Appraisal Center for Environment & Engineering, Ministry of Environmental Protection, Beijing 100012, China

## Abstract

Eucalypt plantations expand rapidly out of their natural distribution zones, thus inducing a concern on their effects on biodiversity and ecosystem functions. We compare the understory plant diversity of 46 plots of eucalypt plantations, including early and later stages in rotation, with that of 21 plots of contrast vegetation, including other types of plantations and secondary shrub grassland, in Guangdong and Guangxi Provinces, South China. Although the overall plant diversity did not change significantly in eucalypt plantations relative to the contrast vegetation, the community structures changed dramatically. The Asteraceae family, which is the most important source of destructive invasive plant species in China, is ranked 3^rd^ (7.42%) and 7^th^ (3.14%) in species importance in the early and later stages in eucalypt plantations, respectively. Nevertheless, Asteraceae is ranked 15^th^ (1.73%) in other types of plantations and 21^st^ (0.94%) in secondary shrub grassland. Significant increases in the richness and frequency of invasive species were also observed in eucalypt plantations. Among the 20 invasive species recorded in the eucalypt plantations, 9 species were destructive invasive species and 7 of these species belonged to Asteraceae. This study highlights an enhanced plant invasion risk in eucalypt plantations in South China, particularly by Asteraceae.

Evidence illustrates that biodiversity contributes to ecosystem functioning^1^,^2^ and that a loss of biodiversity alters ecosystem processes that are essential to the productivity and sustainability of ecosystems^3^,^4^. Natural forests, which provide habitat to the richest terrestrial biodiversity in the world, have been shrinking in size. This environmental change is largely attributed to anthropogenic causes, the most significant of which is deforestation to meet wood supply^5^,^6^,^7^. Plantation forests generally maintain lower biodiversity levels than natural forests^8^,^9^,^10^; however, developing high-yield plantation forests in a sustainable manner may help preserve natural forests and biodiversity.

Eucalypts comprise a group of approximately 800 woody species^11^ that belong to *Eucalyptus, Corymbia* or *Angophora* in Myrtaceae^12^, which are mostly native to Australia but not in China. Eucalypts are among the most fast-growing trees in the world; they adapt well in warm environments and provide materials for timber and pulp^13^. In South China, eucalypt plantations have expanded rapidly in the last decade, from 2.54 M ha in 2008, to 3.6 M ha in 2011^14^ and to 4.40 M ha in 2013^15^. According to the Chinese State Forest Administration, eucalypt plantations in China constituted 22% of the world’s eucalypt plantation area in 2013 and contributed 25% to Chinese wood production^15^. However, despite the remarkable growth of wood production in China, the self-sufficiency rate has declined in recent years (see Supplementary Fig. S1 online) mainly because of the rapid increase in wood consumption. The sustainable development of fast-growing eucalypt plantations is supported by the Chinese government, so as to enhance wood production and to preserve natural forests.

The expansion of eucalypt plantations out of their natural distribution zones has generated concerns about their effects on biodiversity and ecosystem functions. Previous studies have found that the understory plant diversity of eucalypt plantations are important for reducing nutrient loss and soil erosion^16^ and maintaining soil microbial communities^17^; such diversity may have positive effects on the productivity of eucalypts^18^. Eucalypt plantations in South China have been found to maintain a lower diversity of understory plant species than natural forests^9^,^19^. However, other types of plantations, such as *Pinus massoniana* and *Dimocarpus longan* plantations, and secondary shrub grasslands are more common than natural forests in South China, and theses may serve as potential field sources of eucalypt plantations. Whether eucalypt plantations maintain less understory plant diversity than other common types of vegetation remains unclear. Furthermore, previous studies usually focus on diversity indices. However, community structure, such as the relative importance of species or family, typically contains important messages and provides links with the ecological function of community. In another aspect, according to the theory of fluctuating resource availability proposed by Davis *et al.*^20^, the disturbance of understory communities during plantation, fertilization and short rotations of eucalypt plantations may provide opportunities for invasive species to capture light, water and nutrients. Nevertheless, questions like whether eucalypt plantations have more invasive species understory than other common types of vegetation and which environmental factors may affect the invasion risk have been rarely investigated.

We first compare the understory plant diversity, including diversity indices and community structure, in eucalypt plantations with those in other common types of vegetation in South China. Thereafter, we test if more invasive species exist in eucalypt plantations than in contrast vegetation. Finally, we detect the environmental factors that may affect plant invasion in both eucalypt plantations and contrast vegetation.

## Results

### Understory plant diversity of eucalypt plantations

**Figure 1:**
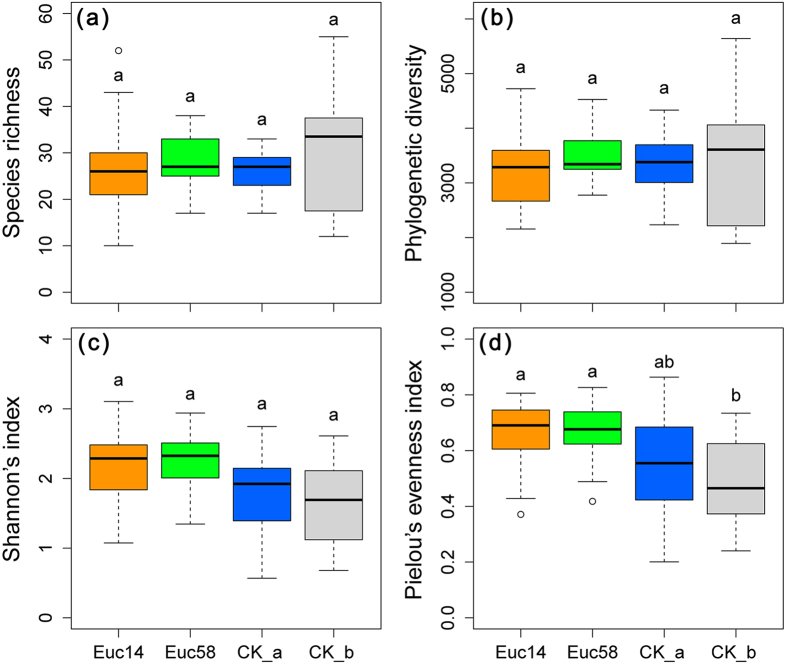
Comparisons of understory plant diversity using species richness (**a**), phylogenetic diversity (**b**), Shannon’s index (**c**) and Pielou’s evenness index (**d**) among two groups of eucalypt plantations (eucalypt plantations with growth years 1–4 (Euc14) and 5–8 (Euc58)) and two groups of contrast vegetation (common types of plantation (CK_a) and secondary shrub grassland (CK_b)). Groups sharing the same letter were insignificantly different at the 95% confidence level by Tukey’s multiple comparisons. For each box-and-whisker plot, the box shows 25% median and 75% quantile of the given values. The whiskers extend to the most extreme data points that are not more than 1.5 times the interquartile range (length of the box) from the box.

We compared species richness, phylogenetic diversity, Shannon’s index and Pielou’s evenness index among four groups, namely, eucalypt plantations with growth year 1–4 (Euc14), eucalypt plantations with growth year 5–8 (Euc58), other types of plantations (CK_a) and secondary shrub grassland (CK_b) by using Tukey’s multiple comparisons (Fig. 1). No significant differences in the aforementioned indices were found among the four groups except for the significant increase in Pielou’s evenness index in the two groups of eucalypt plantations (i.e., Euc14 and Euc58) compared with that in secondary shrub grassland (both P < 0.05).

The 10 most important plant species were identified by averaging the importance values of each species across plots within each of the 4 groups (Table 1). The rank of species importance demonstrated that *Dicranopteris pedata* of Gleicheniaceae was the most important species within each group. However, its importance was lower in eucalypt plantations (16.98% in Euc14 and 19.16% in Euc58) than in contrast vegetation (23.23% in CK_a and 27.61% in CK_b). *Chromolaena odorata* of Asteraceae, an invasive plant species, is ranked 9^th^ in species importance in Euc14. The rank of family importance revealed that the importance of Gleicheniaceae was lower in eucalypt plantations than in contrast vegetation (Table 2). Furthermore, Poaceae and not Gleicheniaceae was the most important family in Euc14. Moreover, the importance of Asteraceae was remarkably higher in eucalypt plantations than in contrast vegetation. Asteraceae ranked 3^rd^ (7.42%), 7^th^ (3.14%), 15^th^ (1.73%) and 21^st^ (0.94%) in Euc14, Euc58, CK_a and CK_b, respectively.

### Diversity of invasive species

**Figure 2:**
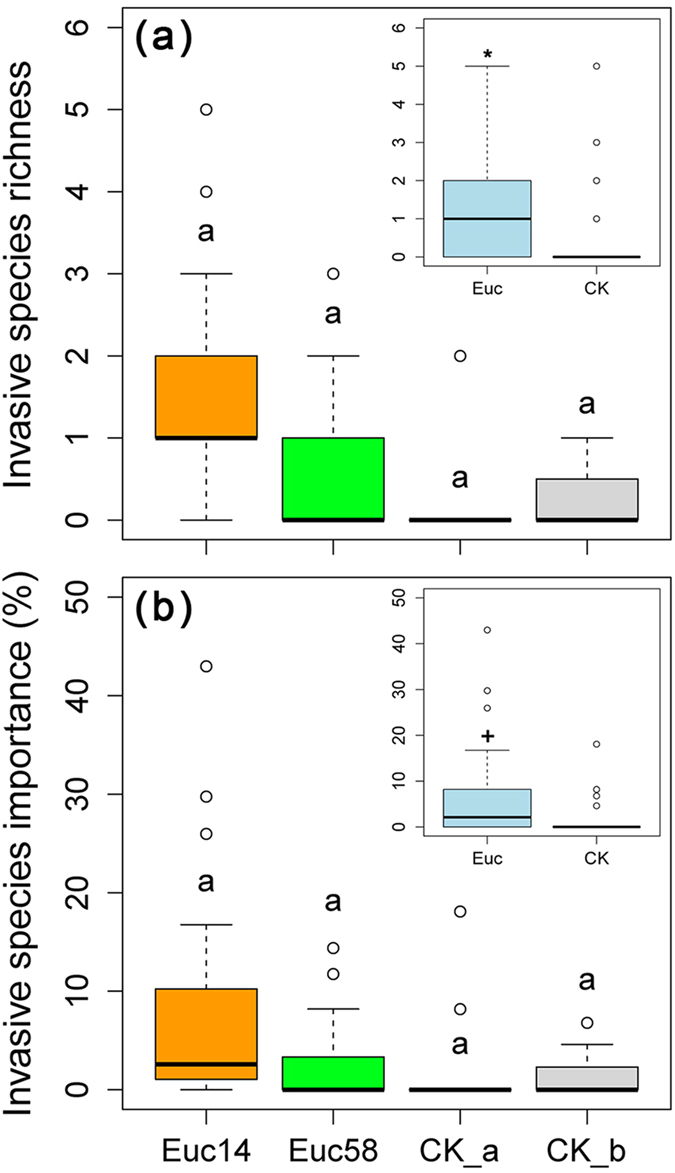
Comparison of invasive species richness (**a**) and importance (**b**) within understory communities between eucalypt plantations and contrast vegetation. Groups sharing the same letter were insignificantly different at the 95% confidence level by Tukey’s multiple comparisons. Euc stands for eucalypt plantations combined with Euc14 and Euc58, and CK stands for contrast vegetation combined with CK_a and CK_b. Significant difference between two groups at the 95% and 90% confidence level were marked with * and ^+^, respectively. The abbreviations of the four groups and description for box-and-whisker plot are the same as in Fig. 1.

Species richness and invasive species importance were compared between eucalypt plantations and contrast vegetation by using Tukey’s multiple comparisons (Fig. 2). Although no significant differences among Euc14, Euc58, CK_a and CK_b were observed, significant increases in both invasive species richness (P < 0.05) and importance (P < 0.10) were noted in the combined eucalypt plantations (Euc) compared with those in the combined contrast vegetation (CK). Moreover, the frequency of invasive species that occurred in a plot was considerably higher in eucalypt plantations than in contrast vegetation: 79.31%, 47.06%, 15.38% and 25.00% in Euc14, Euc58, CK_a and CK_b, respectively.

A total of 20 invasive species were recorded in this study (Table 3), and all of them were noted in eucalypt plantations. *C. odorata, Bidens pilosa* and *Praxelis clematidea* were the 3 major invasive plants in eucalypt plantations; all of which belong to Asteraceae. Among the 9 destructive invasive species recorded, 7 belong to Asteraceae. Moreover, the importance of Asteraceae species among the total importance of invasive plants across plots was higher in eucalypt plantations than in contrast vegetation: 89.45%, 85.91%, 68.81% and 59.86% in Euc14, Euc58, CK_a and CK_b, respectively.

### Environmental factors for plant invasion

Pearson’s correlation between invasive species richness and importance, and 6 environmental factors were performed across 46 plots of eucalypt plantations and 21 plots of contrast vegetation (Table 4). The results indicated that invasive species richness in eucalypt plantations decreased with growth year after plantation (P < 0.05), canopy coverage (P < 0.05) and elevation (P < 0.10) but increased with annual total radiation (P < 0.05) and mean annual temperature (P < 0.1). Invasive species importance exhibited a significant positive correlation with total precipitation per year (P < 0.05). However, no significant correlations were found in contrast vegetation.

## Discussion

Our results revealed that species richness or phylogenetic diversity does not decrease in eucalypt plantations compared with that in contrast vegetation in South China. The high Pielou’s evenness index in eucalypt plantations suggests that the abundance of understory plants was more even among species in eucalypt plantations than in secondary shrub grassland. This finding concurs with the phenomenon that the importance of the dominant species (i.e. *D. pedata*) and Gleicheniaceae family decreases in eucalypt plantations. Considering the biodiversity indices, no reduction in understory diversity was found in eucalypt plantations compared with other common types of vegetation in South China. However, given the change in community structure and the enhanced importance of invasive species, the ecosystem function of eucalypt plantations may have been damaged.

The analysis of community structure illustrated that the importance of the Asteraceae family increase remarkably in eucalypt plantations, particularly in Euc14, which is the early stage of rotation. Moreover, different from contrast vegetation, significant increases in invasive species richness, importance and frequency were observed in eucalypt plantations. Asteraceae comprises a major part in the total number of destructive invasive species and in the total importance of invasive plants in eucalypt plantations. According to a previous research, Asteraceae is the most important source family of invasive plants in China^21^, contributing 52.9% and 18.7% of destructive invasive species and total invasive species in China, respectively^22^,^23^. Hu *et al.*^9^ indicated that *P. clematidea* (under the name *Eupatorium catarium*) and *C. odorata* were important understory species in eucalypt plantations in Hainan Province, China. Our results suggest an enhanced risk of plant invasion in eucalypt plantations in South China, specifically by Asteraceae species.

Furthermore, invasive species richness and importance in the understory are remarkable in Euc14 but tend to decrease in Euc58 as the growth year and canopy coverage increase. This finding concurs with the prediction using the theory of fluctuating resource availability^20^. The causes of the enhanced plant invasion risk in eucalypt plantations, particularly in the early stages by Asteraceae species, may lie in several aspects. First, anthropogenic disturbance during eucalypt plantation often remove small trees and shrubs and reduce the importance of the dominant species understory, thus providing vacant niches for the recruitment of herbaceous plants. Second, Asteraceae species often produce a large quantity of small seeds that are easily dispersed by wind or animals; hence, these species have a good chance to arrive at a vacant niche. Third, eucalypt trees often have narrow leaves hanging vertically in the canopy, thus causing a relatively high light and wind transmittance understory. The increased light availability and soil temperature but low soil water availability under eucalypt plantations may benefit the growth of drought tolerant pioneer plants^24^, such as *C. odorata* and *P. clematidea*. As the eucalypt plantation grows and canopy coverage increases, invasive species gradually lose their advantage against native species in resource competition, particularly with regard to light, and result in their exclusion from the understory community with age of plantation. Besides, our study reveals that eucalypt plantations tend to have an increased number of invasive species when they receive high radiation and precipitation and are located at low elevations; this finding is consistent with the pattern that regions with warm and moist climates tend to have a high number of invasive species in China^21^.

According to our results, to minimize plant invasion risk in eucalypt plantations, we should reduce anthropogenic disturbance of understory community, prolong the rotation length and employ natural succession, which have been reported to promote sustainable eucalypts production as well^16^,^17^,^18^. Asteraceae species, specifically *C. odorata, B. pilosa* and *P. clematidea*, are the most important resource of invasive plants in eucalypt plantations in South China; hence, management should consider their ecological characteristics, including dispersion, phenology and climate thresholds, to minimize the damage they may cause.

## Conclusion

Diversity indices of understory plant community in eucalypt plantations revealed no reduction compared with those of that in other common types of vegetation in South China. However, their community structures differed remarkably. The importance of Asteraceae family was dramatically enhanced in eucalypt plantations, especially in the early stage of rotation. Moreover, increases in species richness, importance and frequency of invasive plant species, mostly of Asteraceae species, were noted in eucalypt plantations rather than in contrast vegetation. Our study highlights an enhanced risk of plant invasion in eucalypt plantations in South China, especially by Asteraceae species. The already vast and continuously expanding area of eucalypt plantations may accelerate native biodiversity loss and ecosystem degradation in South China.

## Methods

### Site description and data collection

**Figure 3:**
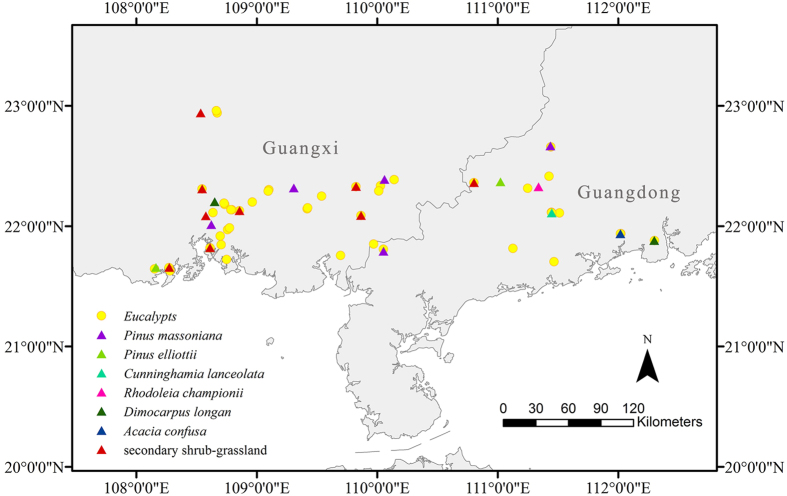
Locations of the 46 plots of eucalypt plantations and 21 plots of 7 types of contrast vegetation investigated in Guangdong and Guangxi Provinces, South China. The map was generated with ArcMap 10.1 with a background of 1: 4,000,000 Administrative Map of the People’s Republic of China^35^.

According to the Eighth National Forest Inventory of China (2009–2013), Guangxi and Guangdong are the 2 provinces with the largest areas of eucalypt plantations; combined, they contributed to 70.5% of eucalypt plantations in China. During 2011 to 2014, we sampled 46 and 21 plots of eucalypt plantations and contrast vegetation, respectively, in Guangdong and Guangxi Provinces in growing season (Fig. 3). The eucalypts were planted with a density of 15–20 individuals per 100 m^2^. The community structure of understory changes during succession after plantation; hence, the eucalypt plantations were grouped into Euc14 (includes 29 plots with growth years 1 to 4) and Euc58 (comprises 17 plots with growth years 5 to 8). The 21 contrast plots representing various common types of vegetation in South China were classified into 2 groups. The first group included 13 plots, covering 6 types of plantations, namely, *P. massoniana, P. elliottii, Cunninghamia lanceolata, Rhodoleia championii, D. longan* and *Acacia confuse*. All of these species are native to China, except for *P. elliottii*. The other group composed of 8 plots was secondary shrub grassland, which was dominated by *D. pedata* of Gleicheniaceae or Poaceae species such as *Miscanthus sinensis* and *Microstegium vimineum*. Overall, the 67 investigated plots ranged from 21°37′44″ to 22°57′37″ N in latitude and from 108°9′10″ to 112°18′7″ E in longitude. According to the Chinese terrestrial ecological information with a resolution of 1 km × 1 km from 1971 to 2000^25^, the annual total solar radiation was 4951 MJ/m^2^, the mean annual temperature was 22.0 °C and the annual total precipitation was 1912 mm in average across the investigated plots.

For each of the 67 plots, we sampled 43 m × 3 m quadrats and recorded the coverage and abundance of each vascular plant species understory. According to Flora of China^26^, 363 species from 229 genera in 85 families were recorded. Invasive plants were confirmed as species with an invasive grade of 1, 2, 3 and 4, which respectively denote destructive, serious, regional and general invasive in the “Checklist of the Chinese Invasive Plants”^23^.

### Statistical analysis

We applied four indices, namely, species richness, phylogenetic diversity^27^, Shannon’s index (equation (1))^28^ and Pielou’s evenness index^29^ (equation (2)), to assess plant diversity in eucalypt plantations and contrast vegetation. To calculate phylogenetic diversity, we constructed a phylogeny tree with all of the recorded species by using the topology of “Phylomatic tree R20120829 for plants” (http://phylodiversity.net/phylomatic/) and adjusted the branch length with the estimated age of important nodes^30^,^31^. The branch length of the phylogeny tree was calculated by using Phylocom (version 4.2)^32^.

*Shannon’s index*


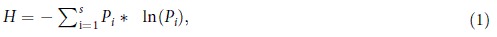


where *P_i_* is the proportion of individuals that belong to the *ith* species in the plot and *s* denotes the total number of species in the same plot.

*Pielou’s evenness index*


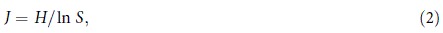


where *H* is Shannon’s index, *S* is the total number of species within a plot and ln (*S*) denotes the maximum value of *H*.

*Importance value*


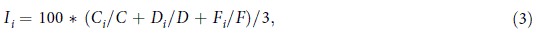


where *C_i_* is the coverage of the *i*th species, *C* is the total coverage of all species within a plot, *D*_*i*_ is the number of the *i*th species, *D* is the total individual number of all species, *F*_*i*_ is the frequency of the *i*th species in the subplots and *F* is the total frequency of all species within a plot.

The importance value of each recorded species within each plot was calculated as the average of relative coverage, relative density and relative frequency in the community (equation (3)). The importance value of each family within each plot was calculated by using a similar method as species importance, with the coverage and number of each family calculated as the sum of species’ values in which each family belongs to. The importance of invasive species within each plot was calculated as the sum of importance for all invasive species within a plot. The diversity indices, importance values and other statistics were calculated by using R version 3.1.2^33^ with the package “vegan” (version 2.2–1)^34^.

## Additional Information

**How to cite this article**: Jin, D. *et al.* High risk of plant invasion in the understory of eucalypt plantations in South China. *Sci. Rep.* **5**, 18492; doi: 10.1038/srep18492 (2015).

## Supplementary Material

Supplementary Figure S1

Supplementary Dataset 1

## Figures and Tables

**Table 1:** Ten most important plant species in eucalypt plantations and contrast vegetation in Guangdong and Guangxi Provinces.

Eucalypt plantations 1–4		Eucalypt plantations 5–8		Other plantations		Secondary shrub grasslands	
Species	Imp	Species	Imp	Species	Imp	Species	Imp
*Dicranopteris pedata*	16.98%	*Dicranopteris pedata*	19.16%	*Dicranopteris pedata*	23.23%	*Dicranopteris pedata*	27.61%
*Miscanthus sinensis*	9.35%	*Miscanthus sinensis*	9.52%	*Microstegium fasciculatum*	7.25%	*Miscanthus sinensis*	7.23%
*Rhodomyrtus tomentosa*	3.36%	*Blechnum orientale*	3.65%	*Miscanthus sinensis*	4.38%	*Microstegium vimineum*	3.79%
*Lygodium microphyllum*	2.81%	*Polygonum chinense*	2.59%	*Blechnum orientale*	3.33%	*Rhodomyrtus tomentosa*	3.11%
*Arthraxon hispidus*	2.38%	*Clerodendrum cyrtophyllum*	2.52%	*Woodwardia japonica*	2.37%	*Imperata cylindrica*	3.10%
*Melastoma dodecandrum*	2.33%	*Rhodomyrtus tomentosa*	2.43%	*Rhodomyrtus tomentosa*	2.35%	*Melastoma dodecandrum*	2.63%
*Blechnum orientale*	2.31%	*Miscanthus floridulus*	2.35%	*Melastoma dodecandrum*	1.76%	*Baeckea frutescens*	2.50%
*Embelia laeta*	2.19%	*Lygodium microphyllum*	2.25%	*Cratoxylum cochinchinense*	1.73%	*Lygodium microphyllum*	1.98%
*Chromolaena odorata*	2.08%	*Microstegium fasciculatum*	1.86%	*Arthraxon hispidus*	1.55%	*Blechnum orientale*	1.95%
*Microstegium fasciculatum*	2.05%	*Arthraxon hispidus*	1.78%	*Pteris semipinnata*	1.38%	*Polygonum chinense*	1.74%

Importance values were averaged across plots within each group.

**Table 2:** Ten most important plant families in eucalypt plantations and contrast vegetation in Guangdong and Guangxi Provinces.

Eucalypt plantations 1–4		Eucalypt plantations 5–8		Other plantations		Secondary shrub grasslands	
Family	Imp	Family	Imp	Family	Imp	Family	Imp
Poaceae	18.90%	Gleicheniaceae	19.47%	Gleicheniaceae	24.75%	Gleicheniaceae	27.61%
Gleicheniaceae	16.98%	Poaceae	18.86%	Poaceae	16.12%	Poaceae	17.06%
Asteraceae	7.42%	Rubiaceae	4.82%	Rubiaceae	5.76%	Myrtaceae	5.90%
Rubiaceae	5.76%	Blechnaceae	3.91%	Blechnaceae	5.70%	Rubiaceae	5.02%
Melastomataceae	4.73%	Verbenaceae	3.85%	Melastomataceae	3.65%	Euphorbiaceae	5.00%
Myrtaceae	4.56%	Euphorbiaceae	3.70%	Pteridaceae	3.35%	Melastomataceae	4.23%
Euphorbiaceae	4.37%	Asteraceae	3.14%	Myrtaceae	3.25%	Lauraceae	2.74%
Lygodiaceae	3.38%	Lygodiaceae	3.12%	Euphorbiaceae	2.95%	Lygodiaceae	2.26%
Primulaceae	2.87%	Myrtaceae	2.98%	Lauraceae	2.91%	Rosaceae	1.99%
Liliaceae	2.74%	Melastomataceae	2.83%	Lygodiaceae	2.46%	Blechnaceae	1.95%

The importance values of the families are shown.

**Table 3:** Invasive species recorded in eucalypt plantations and contrast vegetation, the importance values (Imp) averaged across plots within each group and invasive grade (G).

Eucalypt plantations 1–4			Eucalypt plantations 5–8			Other plantations			Secondary shrub grasslands		
Invasive species	Imp	G	Invasive species	Imp	G	Invasive species	Imp	G	Invasive species	Imp	G
*Chromolaena odorata^ψ^*	2.08%	1	*Bidens pilosa^ψ^*	1.14%	1	*Praxelis clematidea^ψ^*	0.64%	1	*Mimosa bimucronata*	0.57%	1
*Bidens pilos^ψ^*	1.68%	1	*Praxelis clematidea^ψ^*	0.54%	1	*Ageratum conyzoides^ψ^*	0.42%	1	*Bidens pilos^ψ^*	0.52%	1
*Praxelis clematidea^ψ^*	1.12%	1	*Ageratum conyzoides^ψ^*	0.54%	1	*Crotalaria pallida*	0.25%	3	*Erigeron annuus^ψ^*	0.18%	1
*Crassocephalum crepidioides^ψ^*	0.51%	2	*Chromolaena odorata^ψ^*	0.34%	1	*Chromolaena odorata^ψ^*	0.23%	1	*Praxelis clematidea^ψ^*	0.15%	1
*Mikania micrantha^ψ^*	0.38%	1	*Acacia confusa*	0.19%	3	*Panicum maximum*	0.21%	3	**Asteraceae Species**	**0.85%**	
*Panicum maximum*	0.34%	3	*Crotalaria pallida*	0.16%	3	*Cyperus rotundus*	0.17%	4	**sum**	**1.42%**	
*Ageratum conyzoides^ψ^*	0.32%	1	*Solanum torvum*	0.07%	2	*Erigeron canadensis^ψ^*	0.09%	1			
*Erigeron canadensis^ψ^*	0.23%	1	**Asteraceae Species**	**2.56%**		**Asteraceae Species**	**1.39%**				
*Sonchus oleraceus^ψ^*	0.20%	4	**sum**	**2.98%**		**sum**	**2.02%**				
*Erigeron annuus^ψ^*	0.09%	1									
*Alternanthera philoxeroides*	0.07%	1									
*Acacia confusa*	0.06%	3									
*Scoparia dulcis*	0.05%	2									
*Setaria palmifolia*	0.05%	4									
*Oxalis corymbosa*	0.05%	4									
*Cyperus rotundus*	0.05%	4									
*Mimosa bimucronata*	0.04%	1									
*Solanum torvum*	0.04%	2									
*Sida acuta*	0.03%	4									
**Asteraceae Species**	**6.62%**										
**sum**	**7.40%**										

Species belong to Asteraceae family were marked with ψ.

**Table 4:** Pearson’s correlations between invasive species richness, importance and environmental factors across 46 plots of eucalypt plantations and across 21 plots of contrast vegetation.

Environmental factors	Eucalypt plantations		Contrast vegetation	
	Richness	Importance	Richness	Importance
Canopy coverage	**−0.294**, 0.050	−0.184, 0.227	−0.381, 0.222	−0.357, 0.255
Growth year after plantation	***−*****0.352**, 0.024	−0.152, 0.341	—	—
Elevation	−*0.270*, 0.070	−0.199, 0.186	−0.135, 0.560	−0.122, 0.560
Annual total radiation	**0.309**, 0.037	0.200, 0.184	0.264, 0.248	0.293, 0.197
Mean annual temperature	*0.285*, 0.055	0.186, 0.217	0.245, 0.285	0.254, 0.266
Annual total precipitation	0.136, 0.368	**0.301**, 0.042	−0.081, 0.727	−0.126, 0.588

The correlation coefficients and P values are shown; coefficients with P values < 0.05 are in bold and those with P values < 0.1 are in italic. Symbol “—” means data not available.
