# Supplementary material for: High risk of plant invasion in the understory of eucalypt plantations in South China
Source: Sci Rep. 2015 Dec 21;5:18492. doi: 10.1038/srep18492 (PMC4685264; doi:10.1038/srep18492)
Supplement: Supplementary Figure S1 [file srep18492-s1.pdf]

Supplementary Information for:

High risk of plant invasion in the understory of eucalypt plantations in South China

Dongmei Jin[1#], Yong Huang[2#], Xile Zhou[1], Bin Chen[1], Jinshuang Ma[1], Yuehong Yan[1*]

1 Shanghai Chenshan Plant Science Research Center, Chinese Academy of Sciences /Shanghai Chenshan Botanical Garden, Shanghai 201602, China

2 Appraisal Center for Environment and Engineering, Ministry of Environmental Protection, Beijing 100012, China

[#] The authors contributed equally to this article.

* Corresponding author, e-mail: yhyan@sibs.ac.cn, tel: (86) 021-37792288-903, address: 3888 Chenhua Road, Songjiang, Shanghai 201602, China.

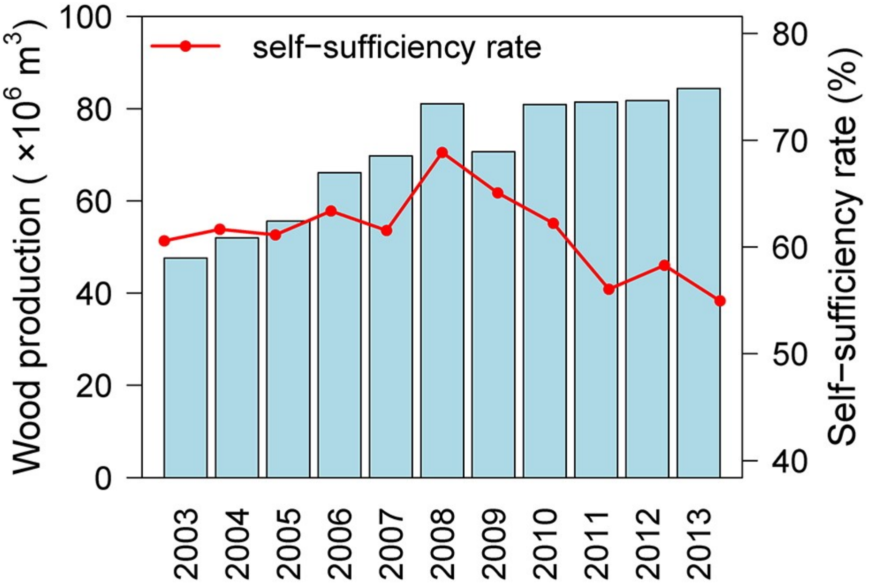

Figure S1. Chinese wood production and self-sufficiency rate during years 2003-2013. Data were compiled from National Bureau of Statistics of China[36].

36 National Bureau of Statistics of China. *National data.* Available at: http://data.stats.gov.cn (Accessed: 12th April 2015).
